# Supplementary material for: High-flow nasal cannula for reducing hypoxemic events in patients undergoing bronchoscopy: A systematic review and meta-analysis of randomized trials
Source: PLoS One. 2021 Dec 1;16(12):e0260716. doi: 10.1371/journal.pone.0260716 (PMC8635390; doi:10.1371/journal.pone.0260716)
Supplement: S1 Appendix — (DOCX) [file pone.0260716.s001.docx]

| S1 Appendix: Search Strategy | | |
| --- | --- | --- |
| Search | Query | Items found |
|  | Pubmed (November 06, 2021) |  |
| #10 | #6 AND #9 | 105 |
| #9 | #7 OR #8 | 41561 |
| #8 | Bronchoscope | 12663 |
| #7 | Bronchoscopy | 36708 |
| #6 | #1 OR #2 OR #3 OR #4 OR #5 | 16911 |
| #5 | High flow oxygen | 16131 |
| #4 | High flow nasal oxygen therapy | 1508 |
| #3 | High flow nasal oxygen | 1879 |
| #2 | High flow nasal cannula | 2145 |
| #1 | HFNC | 800 |
|  | Embase (November 06, 2021) |  |
| #9 | #4 AND #8 | 178 |
| #8 | #5 OR #6 OR #7 | 76578 |
| #7 | ‘Bronchoscope’/exp OR ‘bronchoscope’ | 10047 |
| #6 | ‘Fiberoptic bronchoscopy’/exp OR ‘fiberoptic bronchoscopy’ | 11835 |
| #5 | ‘Bronchoscopy’/exp OR ‘bronchoscopy’ | 72937 |
| #4 | #1 OR #2 OR #3 | 3185 |
| #3 | ‘High flow oxygen therapy’/exp OR ‘high flow oxygen therapy’ | 2877 |
| #2 | ‘High flow nasal cannula oxygen therapy’/exp OR ‘high flow nasal cannula oxygen therapy’ | 308 |
| #1 | ‘High flow nasal cannula therapy’/exp OR ‘high flow nasal cannula therapy’ | 2642 |
|  | Cochrane library (November 06, 2021) |  |
| #13 |  |  |
| #12 | #6 AND #11 | 75 (55 trials) |
| #11 | #7 OR #8 OR #9 OR #10 | 2881 |
| #10 | MeSH descriptor: [Bronchoscopes] explode all trees | 88 |
| #9 | Bronchoscope | 766 |
| #8 | MeSH descriptor: [Bronchoscopy] explode all trees | 733 |
| #7 | Bronchoscopy | 2529 |
| #6 | #1 OR #2 OR #3 OR #4 OR #5 | 4127 |
| #5 | High flow oxygen | 3827 |
| #4 | High flow nasal oxygen therapy | 1126 |
| #3 | High flow nasal oxygen | 1453 |
| #2 | High flow nasal cannula | 1159 |
| #1 | HFNC | 569 |

The list of 24 major exclusions with references were reported as below:

1. Non-RCTs (n = 18)
2. Case report [1–6]

(2) Conference abstract [7–11]

(3) Editorial or comments [12–14]

(4) Observational studies [15–18]

1. Different comparison (n = 2) [19,-20]
2. Non-adult (n = 2) [21,22]
3. Indication for intubation (n = 2) [22–24]

1. Berlin D, Singh I, Barjaktarevic I, Friedman O. A Technique for Bronchoscopic Intubation During High-Flow Nasal Cannula Oxygen Therapy. J Intensive Care Med. 2016;31(3): 213-215. DOI: [10.1177/0885066615582020](https://doi.org/10.1177/0885066615582020).

2. Diab S, Fraser JF. Maintaining Oxygenation Successfully with High Flow Nasal Cannula during Diagnostic Bronchoscopy on a Postoperative Lung Transplant Patient in the Intensive Care. Case Rep Crit Care. 2014;2014: 198262. DOI: [10.1155/2014/198262](https://doi.org/10.1155/2014/198262).

3. Kurose H, Seki H, Ideno S, Kato J, Morisaki H. High-flow nasal cannula oxygenation during interventional bronchoscopy in a patient with severe tracheal stenosis. J Clin Anesth. 2018;46: 92-93. DOI: [10.1016/j.jclinane.2018.02.008](https://doi.org/10.1016/j.jclinane.2018.02.008).

4. Min JY, Jo H, Roh K, Chung MY. Preventing deoxygenation with high flow nasal cannula oxygen during induction of general anesthesia for rigid bronchoscopy: Two case reports. Medicine. 2019;98(27): e15998. DOI: [10.1097/MD.0000000000015998](https://doi.org/10.1097/MD.0000000000015998).

5. Pb S, Hadda V, Madan K, Tiwari P, Mohan A. Flexible Bronchoscopy in a Patient With Pneumomediastinum and Hypoxemic Respiratory Failure: High-Flow Nasal Cannula to the Rescue. Cureus. 2021;13(1): e12800. DOI: [10.7759/cureus.12800](https://doi.org/10.7759/cureus.12800).

6. Thoker ZA, Hadda V, Tiwari P, Mittal S, Madan K, Mohan A. High-flow nasal cannula for oxygenation during emergency tracheal stenting under flexible bronchoscopy guidance. Lung India. 2021;38(3): 269-272. DOI: [10.4103/lungindia.lungindia_395_20](https://doi.org/10.4103/lungindia.lungindia_395_20).

7. Cobos J, Ledesma G, Tirape H, Jaramillo B, Ruiz J, Pacheco L, et al. Comparative study of two modalities of oxygen therapy during flexible bronchoscopy in critically ill patients: High-flow nasal oxygen therapy vs conventional oxygen therapy. Intensive Care Med Exp. 2020;8(suppl 2).

8. Hersch N, Don G, Hibbert M, Mellish C, Ottey J, Newman J, et al. A comparison of high flow versus standard low flow nasal oxygen during diagnostic bronchoscopy. Respirol Conference: 2016 annual scientific meetings of the thoracic society of australia and new zealand and the australian and new zealand society of respiratory science, TSANZSRS 2016 Perth, WA Aust Conference start: 20160401 Conference end: 20160406 Conference publication: (varpagings);21; 2016. p. 70.

9. Hong G, Kim DH, Kim YS, Park JS, Jee YK. Usefulness of high-flow nasal cannula oxygen therapy in patients with acute respiratory failure undergoing bronchoalveloar lavage. Intensive Care Med Exp. 2016;4.

10. Lin CW, Lin TY, Lo YL. The impact of the high-flow nasal oxygen to the safety and sedative quality of patients undergoing bronchoscopy-preliminary data. Respirology. Carlton, Vic. 2018;23: 104–105. DOI: [10.1111/resp.13420_39](https://doi.org/10.1111/resp.13420_39).

11. Park Y, Kim Y, Park J, Kim ES, Lee SH, Lee YJ, et al. High Flow nasal OXygen therapy in high risk patients of hypoxia undergoing diagnostic BRONCHOscopy (HiFOXyBRONCHO): a prospective, randomized, controlled study. Eur Respir J. 2018;52.

12. La Combe B, Messika J, Fartoukh M, Ricard JD. Increased use of high-flow nasal oxygen during bronchoscopy. Eur Respir J. 2016;48(2): 590-592. DOI: [10.1183/13993003.00565-2016](https://doi.org/10.1183/13993003.00565-2016).

13. Nanavaty P, Feller-Kopman DJ. Go with the flow? High flow nasal cannula for bronchoscopy. Thorax;15;thoraxjn1-2021-217450. DOI: [10.1136/thoraxjnl-2021-217450](https://doi.org/10.1136/thoraxjnl-2021-217450).

14. Santos PS, Cruz C, Esquinas AM. Increased use of high-flow nasal oxygen during bronchoscopy. Eur Respir J. 2016;48(2): 589. DOI: [10.1183/13993003.00441-2016](https://doi.org/10.1183/13993003.00441-2016).

15. Chung SM, Choi JW, Lee YS, Choi JH, Oh JY, Min KH, et al. Clinical Effectiveness of High-Flow Nasal Cannula in Hypoxaemic Patients during Bronchoscopic Procedures. Tuberc Respir Dis (Seoul). 2019;82(1): 81-85. DOI: [10.4046/trd.2017.0104](https://doi.org/10.4046/trd.2017.0104).

16. Kim EJ, Jung CY, Kim KC. Effectiveness and Safety of High-Flow Nasal Cannula Oxygen Delivery during Bronchoalveolar Lavage in Acute Respiratory Failure Patients. Tuberc Respir Dis (Seoul). 2018;81(4): 319-329. DOI: [10.4046/trd.2017.0122](https://doi.org/10.4046/trd.2017.0122).

17. La Combe B, Messika J, Labbé V, Razazi K, Maitre B, Sztrymf B, et al. High-flow nasal oxygen for bronchoalveolar lavage in acute respiratory failure patients. Eur Respir J. 2016;47(4): 1283-1286. DOI: [10.1183/13993003.01883-2015](https://doi.org/10.1183/13993003.01883-2015).

18. Service JA, Bain JS, Gardner CP, McNarry AF. Prospective Experience of High-flow Nasal Oxygen During Bronchoscopy in 182 Patients: A Feasibility Study. J Bronchology Interv Pulmonol. 2019;26(1): 66-70. DOI: [10.1097/LBR.0000000000000533](https://doi.org/10.1097/LBR.0000000000000533).

19. Saksitthichok B, Petnak T, So-Ngern A, Boonsarngsuk V. A prospective randomized comparative study of high-flow nasal cannula oxygen and non-invasive ventilation in hypoxemic patients undergoing diagnostic flexible bronchoscopy. J Thorac Dis. 2019;11(5): 1929-1939. DOI: [10.21037/jtd.2019.05.02](https://doi.org/10.21037/jtd.2019.05.02).

20. Simon M, Braune S, Frings D, Wiontzek AK, Klose H, Kluge S. High-flow nasal cannula oxygen versus non-invasive ventilation in patients with acute hypoxaemic respiratory failure undergoing flexible bronchoscopy--a prospective randomised trial. Crit Care. 2014;18(6): 712. DOI: [10.1186/s13054-014-0712-9](https://doi.org/10.1186/s13054-014-0712-9).

21. Caruso TJ, Gupta A, Sidell DR, Darling C, Rodriguez S, Fonseca A, et al. The successful application of high flow nasal oxygen during microdirect laryngoscopy and bronchoscopy in patients under 7 kg. J Clin Anesth. 2019;52: 27-28. DOI: [10.1016/j.jclinane.2018.08.027](https://doi.org/10.1016/j.jclinane.2018.08.027).

22. Sharluyan A, Osona B, Frontera G, Brandstrup KB, Figuerola J, Sanz-Ruiz I, et al. High flow nasal cannula versus standard low flow nasal oxygen during flexible bronchoscopy in children: A randomized controlled trial. Pediatr Pulmonol. 2021. Sep 10: 1-10. doi: [10.1002/ppul.25655](https://doi.org/10.1002/ppul.25655).

23. Lee MH, Kim HJ. Application of high-flow nasal oxygenation as a rescue therapy in difficult videolaryngoscopic intubation. SAGE Open Med Case Rep. 2021;9: 2050313X211010015. DOI: [10.1177/2050313X211010015](https://doi.org/10.1177/2050313X211010015), [2050313](http://www.ncbi.nlm.nih.gov/pubmed/2050313).

24. Zou T, Huang Z, Hu X, Cai G, He M, Wang S, et al. Clinical application of a novel endoscopic mask: a randomized controlled, multi-center trial in patients undergoing awake fiberoptic bronchoscopic intubation. BMC Anesthesiol. 2017;17(1): 79. DOI: [10.1186/s12871-017-0370-y](https://doi.org/10.1186/s12871-017-0370-y).
