# Supplementary figures and images for: High-flow nasal cannula for reducing hypoxemic events in patients undergoing bronchoscopy: A systematic review and meta-analysis of randomized trials
Source: PLoS One. 2021 Dec 1;16(12):e0260716. doi: 10.1371/journal.pone.0260716 (PMC8635390; doi:10.1371/journal.pone.0260716)

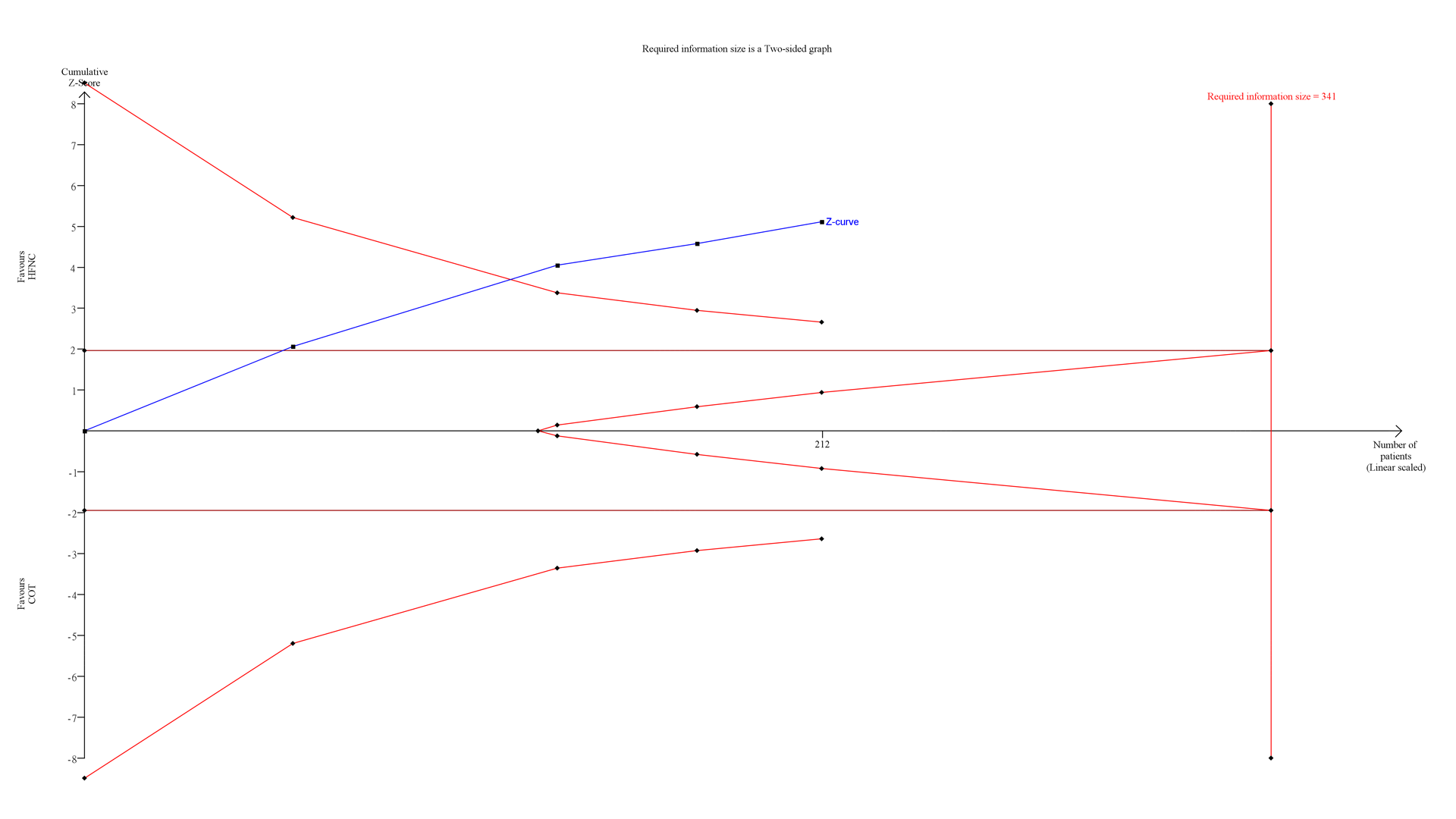

Supplement: S3 Appendix — (TIF) [file pone.0260716.s003.tif]
