# Supplementary material for: High-flow nasal cannula for reducing hypoxemic events in patients undergoing bronchoscopy: A systematic review and meta-analysis of randomized trials
Source: PLoS One. 2021 Dec 1;16(12):e0260716. doi: 10.1371/journal.pone.0260716 (PMC8635390; doi:10.1371/journal.pone.0260716)
Supplement: S4 Appendix — (DOCX) [file pone.0260716.s004.docx]

In our meta-analysis, we enrolled one trial that included patients with lung transplantation undergoing bronchoscopy. To examine whether this trial would influence our findings, we performed sensitivity analyses accordingly. The result of the sensitivity analysis was similar to that of our previous data synthesis. The syntheses for incidence of hypoxemic events (oxygen saturation [SpO_2_] < 90%) during bronchoscopy, incidence of interrupted bronchoscopy due to desaturation, lowest oxygen saturation (SpO_2_) during bronchoscopy, partial pressure of oxygen (PaO_2_), partial pressure of carbon dioxide (PaCO_2_), and end-tidal CO_2_ (EtCO_2_) at the end of bronchoscopy are described below:

| **S3 Appendix: Sensitivity analysis** | | | | | | |
| --- | --- | --- | --- | --- | --- | --- |
| Outcome | No. of studies | Effect size  (95% CI) before sensitivity analysis | Heterogeneity | No. of studies | Effect size  (95% CI) after sensitivity analysis | Heterogeneity |
| Incidence of hypoxic events (SpO_2_ < 90%) | 4 | RR: 0.25  (0.14−0.42) | 0% | 3 | RR: 0.26  (0.12−0.55) | 1% |
| Incidence of interrupted bronchoscopy due to desaturation | 2 | RR: 0.19  (0.02−1.86) | 37% | 1 | RR: 0.50  (0.05−5.22) | NA |
| Lowest SpO_2_ during bronchoscopy | 4 | WMD: 7.12  (5.39–8.84) | 43% | 3 | WMD: 6.85  (4.58–9.13) | 57% |
| PaO_2_ at the end of bronchoscopy | 2 | WMD: 20.36  (0.30–40.42) | 41% | 2 | WMD: 20.36  (0.30–40.42) | 41% |
| PaCO_2_ at the end of bronchoscopy | 2 | WMD: −0.02  (−2.31–2.27) | 22% | 2 | WMD: −0.02  (−2.31–2.27) | 22% |
| EtCO_2_ at the end of bronchoscopy | 2 | WMD: −0.12  (−4.19–3.94) | 21% | 2 | WMD: −0.12  (−4.19–3.94) | 21% |
| Abbreviations: CI, confidence interval; NA, not applicable; RR, risk ratio; WMD, weighted mean difference | | | | | | |
