# Supplementary material for: High-flow nasal cannula for reducing hypoxemic events in patients undergoing bronchoscopy: A systematic review and meta-analysis of randomized trials
Source: PLoS One. 2021 Dec 1;16(12):e0260716. doi: 10.1371/journal.pone.0260716 (PMC8635390; doi:10.1371/journal.pone.0260716)
Supplement: Attachment — Submitted filename: Response to Reviewers.docx [file pone.0260716.s005.docx]

Ref: PONE-D-21-21235

Title: High-flow nasal cannula for reducing hypoxemic events in patients receiving bronchoscopy: A systematic review and meta-analysis of randomized trials.

Dear Editors and Reviewers of *PLOS ONE*:

We sincerely appreciate your review of our manuscript entitled " High-flow nasal cannula for reducing hypoxemic events in patients undergoing bronchoscopy: A systematic review and meta-analysis of randomized trials." We hope that our edits and the responses we provide below satisfactorily address all issues and concerns you and the reviewers have noted and that the revised manuscript will now be suitable for publication in your journal.

Sincerely Yours,

RRT. Ming-Chi Hu

Taipei Medical University

**Statistical Editor:**
1. Please ensure that your manuscript meets PLOS ONE's style requirements, including those for file naming. The PLOS ONE style templates can be found at https://journals.plos.org/plosone/s/file?id=wjVg/PLOSOne_formatting_sample_main_body.pdf and https://journals.plos.org/plosone/s/file?id=ba62/PLOSOne_formatting_sample_title_authors_affiliations.pdf
2. PLOS requires an ORCID iD for the corresponding author in Editorial Manager on papers submitted after December 6th, 2016. Please ensure that you have an ORCID iD and that it is validated in Editorial Manager. To do this, go to ‘Update my Information’ (in the upper left-hand corner of the main menu), and click on the Fetch/Validate link next to the ORCID field. This will take you to the ORCID site and allow you to create a new iD or authenticate a pre-existing iD in Editorial Manager. Please see the following video for instructions on linking an ORCID iD to your Editorial Manager account: https://www.youtube.com/watch?v=_xcclfuvtxQ
Response: Thank you for your suggestion. We have ensured that our manuscript meets the style requirements of *PLOS ONE* and have provided the ORCID ID in the Editorial Manager as required.

Additional Editor Comments :
A number of issues have been identified in the review process. While we feel that this manuscript shows promise, we also think that a major revision is needed. Before we can make a final decision about this manuscript we want to offer you the opportunity to revise and resubmit the manuscript.

1- Methods. Please include the new PRISMA 2020 checklist in the supplementary material.
Response: Thank you for your suggestion. We have provided the new PRISMA 2020 checklist.

2- Methods. The 4 search strategies should be reported in the supplementary material.
Response: Thank you for your suggestion. The search strategies have been provided in the supplementary material (S1 Appendix) accordingly.

3- Methods. How did you manage missing outcome data? Did you contact corresponding authors?
Response: Thank you for your suggestion. When preparing this manuscript, we closely examined the missing outcome data. Because the authors provided a rationale for any missing outcome data, we did not contact corresponding authors directly for this purpose.

4- Methods. You should consider to perform a trial sequential analysis for the primary outcome to assess risk of type 1 and 2 errors.
Response: Thank you for your suggestion. We tried to perform a trial sequential analysis (TSA) to the extent possible. The results of this analysis showed that the Z-curve crosses the O’Brien-Fleming boundaries after the fifth cumulative significance testing. Conclusions made using TSA may indicate that HFNC would reduce the hypoxemic events (SpO_2_ < 90%) in patients undergoing bronchoscopy. The results of the TSA remained significant. We have provided the corresponding results in the fourth paragraph of the Results section and in the supplementary material (S2 Appendix).

5- Results. The list of 27 major exclusions should be reported in the supplement (reference + reason for exclusion).
Response: Thank you for your suggestion. We have provided the list of major exclusions in supplementary material (S1 Appendix) as required.

6- Results. Instead of Figure 1, please use a PRISMA 2020 flow-chart.
Response: Thank you for your suggestion. We have updated the literature research and have provided a PRISMA 2020 flow chart accordingly.

7- Results. Systematic searches found only 157 records. This is correct?
Response: Thank you for your suggestion. We have updated the relevant trials published before August 2021 and have provided relevant information in the first paragraph of the Results, Fig 1, and supplementary material (S1) as required.

8- Results. You reported that all trials had low risk in the reporting bias item. Do you confirm that all trials had a registered protocol?
Response: Thank you for your suggestion. To assess the reporting bias, we closely reviewed the protocols in ClinicalTrial.gov. Although Irfan et al. and Lucangelo et al. did not provide information on registration, they still investigated the outcomes that concerned other registrators. Hence, we did not change our assessment. To clarify this issue, we have added the corresponding rationale to Table 2.

9- Results. You reported that all trials had low risk in performance bias. Do you confirm that all studies had ITT analysis?
Response: Thank you for your suggestion. First, there was one patient who crossed over from the conventional oxygen group to the HFNC group in Ben-Menachem’s trial, but they performed adequate analysis on an intend-to-treat (ITT) basis. Second, although it was difficult for the research to be double blind, no obvious deviations from the intended interventions were detected. Hence, we did not raise the risk of bias. To clarify this issue, we have added the corresponding rationale to Table 2.

10- Results. Methodology on GRADE should be moved to the Methods.
Response: Thank you for your suggestion. Because the GRADE methodology was described in the Methods section, we have deleted the description of the GRADE methodology from the ninth paragraph of the Results section.

11- Results. The GRADE assessment for the primary outcome and lowest SpO2 had no serious concern on imprecision. However, only 4 RCTs reported the outcome.
Response: Thank you for your suggestion. Regarding the incidence of hypoxemic events (SpO2 < 90%) 2, the 95% confidence interval (CI) was short, no effects were excluded, and there was no overlap with the risk ratio of 0.75. Thus, we considered that the effect size was precise. Besides, we also utilized trial sequential analysis to support our findings. Regarding the lowest SpO_2_, the effect size was satisfactory and the power was sufficient. In this case, we considered that the concept was similar with this reference (PMID:30545967). Because the estimator was significant at 7.12%, and the 95% CI did not exclude no effects, we did not raise the uncertainty.

12- Results. The studies were performed under local anesthesia? Sedation?
Response: Thank you for your suggestion. We have provided this information in the seconded paragraph of the Results section and in Table 1.

13- Discussion. The section should be tempered; GRADE results should be considered.
Response: Thank you for your suggestion. We have revised the first paragraph of the Discussion section also considering the GRADE results.

14- Discussion. The inclusion/exclusion criteria of the RCTs limit the external validity of the results. Please discuss.
Response: Thank you for your comment. Because we followed the narrowed PICO selection, there would be a limitation regarding external validity. We have added this limitation to the seventh paragraph of the Discussion section accordingly.

15- Abstract. Conclusions of the abstract should be tempered since the certainty of evidence is moderate/low.
Response: Thank you for your suggestion. We have revised the Abstract and Conclusion sections as required.

Please use the space provided to explain your answers to the questions above. You may also include additional comments for the author, including concerns about dual publication, research ethics, or publication ethics. (Please upload your review as an attachment if it exceeds 20,000 characters)

Reviewer #1: I have reviewed the manuscript that is interesting. However, there are some limitation or concerns requiring discussion.

Comments:
1. English requires an extensive revision by a mother language author or service.
Response: Thank you for your suggestion. We acknowledge Editage Academic Editing for editing this manuscript after the major revision.

2. Intro: “Sedation and the occlusion of the bronchi during the procedure reduce the respiratory drive and alter the lung condition.” The reviewer agree that sedation may alter the respiratory drive or timing (refer to PMID: 23982026 and 28673877); however, it remains unclear what authors mean for “lung condition”. Please specify.
Response: Thank you for your suggestion. We have cited PMID: 23982026 and 28673877 as required.

3. Intro: this section requires more quotation to support all statements.
Response: Thank you for your suggestion. We have provided the references (PMID: 23982026, 28673877, PMID: 30725265, PMID: 25498581, PMID: 25742321, PMID: 27856714, and PMID: 26329355) in the Introduction and Discussion sections as required.

4. Aim of the study: “we compared the efficacy and safety of HFNC with conventional oxygen”. Please specify what is efficacy and safety, even in the material and methods section
Response: Thank you for your suggestion. We have revised the first and second paragraphs of the Introduction section and the seventh paragraph of the Methods section. We have also provided references to support our reasoning.

5. I would suggest to add a “take-home message” regarding possible indication on the use of HFNC over COT during bronchoscopy.
Response: Thank you for your suggestion. First, the number of relevant trials regarding HFNC in high-risk patients undergoing bronchoscopy was not sufficient to make robust indications. Second, we did not make decisive instruction to make our manuscript more tempered according to reviewer’s suggestions. However, we still reviewed relevant references and have provided possible indications and limitations in the fourth and seventh paragraphs of the Discussion section.

Reviewer #2: The study is useful for end-users and the process is well-conducted. The PICO selection should appear in the method section.

Introduction: well balanced
Method: appropriate
Results: standard
Discussion: moderately interesting
Conclusion: based on results
Illustrations: standard
Response: Thank you for your suggestion. We have added information on the PICO selection to the first paragraph of the Methods section per your request.

Reviewer #3: This is a registered systematic review & meta-analysis of five RCTs that aimed to evaluate whether high flow therapy (HFT) is more effective at preventing hypoxemic events during bronchoscopy. It is a well written manuscript that addresses an important clinical area, however a number of issues must be addressed prior to publication.

Major:
- A definition of hypoxemic events must be provided in the methods and referred to throughout the results and discussion, with a clear justification for the chosen definition. Without this, an ambiguous reference to hypoxemic events does not have clinical relevance and cannot contribute meaningfully to the evidence base.
Response: Thank you for your suggestion. We have defined “hypoxemic events” in the second paragraph of the Abstract, the second paragraph of the Introduction section, the fourth and ninth paragraphs of the Results section, Table 3, and the first, third, and fourth paragraphs of the Discussion section as requested.

- Descriptions of physiological effects of HFNC are clear and useful, however no references have been provided for each effect. Please refer to the relevant clinical and bench studies for each point made (consider reviewing PMID: 33664838 for guidance).
Response: Thank you for your suggestion. We have cited PMID 33664838 as requested.

- Ambiguous references to potential adverse events during/following bronchoscopy (in the introduction and discussion) should be replaced with specific outcomes and references.
Response: Thank you for your suggestion. We have reviewed PMID: 25498581, PMID: 23982026, and PMID: 28673877 accordingly.

Minor:
Grammatical and style suggestions have been provided below, which would aid clarity.
ABSTRACT
- Line 26 Suggest “undergoing” rather than “receiving” bronchoscopy
Response: Thank you for your suggestion. We have replaced “receiving” with “undergoing” throughout the manuscript.

- Use comma not semi-colon when listing results
Response: Thank you for your suggestion. We have replaced the semicolons with commas in the Abstract and the fifth paragraph of the Methods section.

INTRODUCTION
- First two sentences too ambiguous, need to provide more detail on potential complications of bronchoscopy (remove “respiratory disturbance” and “alter the lung condition”).
Response: Thank you for your suggestion. We have revised the first paragraph of the Introduction and Conclusion sections and have cited PMID: 25498581, PMID: 23982026, and PMID: 28673877 to describe the potential complications of bronchoscopy.

- There is not “usually” a high risk of hypoxemic events requiring intervention – this is rare during elective procedures.
Response: Thank you for your suggestion. We have replaced “usually” with “occasionally” in the first paragraph of the Introduction section.

- What evidence is there that hypoxaemia and respiratory distress are debilitating? Please provide references.
Response: Thank you for your suggestion. We have revised the first paragraph of the Introduction section and have provided references as requested.

- Line 69 – cardiothoracic surgeons may also perform bronchoscopy, remove “physicians”
Response: Thank you for your suggestion. We have revised the second paragraph of the Introduction section as requested.

- “Although oxygen therapy…” is a nebulous statement, remove or make more specific with dates and a reference.
Response: Thank you for your suggestion. We have revised the second paragraph of the Introduction section as requested.

- Conventional oxygen does not have limited effectiveness (line 73), the point here is that HFNC may be more effective.
Response: Thank you for your suggestion. We have revised the second paragraph of the Introduction section as requested.

- HFNC has many, many more applications than treating hypoxaemic respiratory failure (lines 77-78). Either remove statement or provide specific applications (summarised in aforementioned review PMID: 33664838)
Response: Thank you for your suggestion. We have cited PMID: 33664838 and have revised the third paragraph of the Introduction as requested.

- Do not reference one article for its physiological effects, individual trials need to be referenced for each point.
Response: Thank you for your suggestion. We have cited the following studies (PMID: 30725265, PMID: 25498581, PMID: 25742321, PMID: 27856714, and PMID: 26329355) to support each effect and have revised the third paragraph of the Introduction section as requested.

- Line 82 – not strictly true, the Service paper referenced, which is a feasibility study, did report conclusive results.
Response: Thank you for your suggestion. We have revised the third paragraph of the Introduction section according to the reviewer’s comment.

- Rephrase line 85 “Thus, through a SR…” to “The aim of this systematic review was to…” and list aims.
Response: Thank you for your suggestion. We have revised the third paragraph of the Introduction section as requested.

METHODS
- Inclusion criterion 3 is unclear – what is meant by “clearly” reporting eligibility criteria? Or medical treatments regimens or severity among the population? Needs to be more specific.
Response: Thank you for your suggestion. We have added information on the PICO selection to the first paragraph of the Methods section per your request.

- Hypoxic events must be given a clear definition, particularly since this is the primary aim of the systematic review.
Response: Thank you for your suggestion. We have defined “hypoxemic events” in the second paragraph of the Abstract, the second paragraph of the Introduction section, the fourth and ninth paragraphs of the Results section, Table 3, and the first, third, and fourth paragraphs of the Discussion section as requested.

- Outcomes – use commas, not semicolons
Response: Thank you for your suggestion. We have replaced the semicolons with commas in the Abstract and the fifth paragraph of the Methods section.

RESULTS
- What are the irrelevant trials? This needs to be more specific.
Response: Thank you for your suggestion. We have revised the first paragraph of the Results section to include the PICO selection and have added the relevant rationale in detail.

- Line 169 “recruited patients of middle-to-old age” is not appropriate. Either provide the mean ± SD / median (IQR) age or omit.
Response: Thank you for your suggestion. We have revised the second paragraph of the Results section accordingly.

- Line 169 – what is the relevance of including how studies reported baseline oxygenation? In the results, you describe that 4 studies use SpO2.
Response: Thank you for your suggestion. We considered that the accuracy of SpO_2_, PaO_2,_ and PaO_2_/FiO_2_ ratio were different. To emphasize these differences, and examine the baseline characteristics in a proper way, we have summarized the relevant oxygen indicators in the second paragraph of the Results section and in Table 1.

- Reference 12 is a very specific population at significantly higher risk of hypoxaemia/adverse events – this needs to be commented on.
Response: Thank you for your suggestion. We have expanded the second paragraph of the Results section and the fourth paragraph of the Discussion section accordingly.

- The allocation bias to malignancy is surely acceptable given this procedure is commonly performed to investigate suspected cancer?
Response: Thank you for your suggestion. According to the inclusion criteria and Table 1 in each trial, no relevant information regarding suspected cancer was provided. Because it limited the external validity of the results, we have added this limitation to the seventh paragraph of the Discussion section.

- Risk of bias needs to be reported as low, high or unclear, not “Some concerns” (see Cochrane guidance), or the “some” needs to be elaborated on.
Response: Thank you for your suggestion. Low, high, or unclear would be used in risk-of-bias tool, version 1. However, we utilized risk-of-bias tool, version 2 as our appraisal tool (https://training.cochrane.org/handbook/current/chapter-08).

- Line 190 – hypoxic events has not been defined. This must be described in the methods for these analyses to have any relevance.
Response: Thank you for your suggestion. We have defined “hypoxemic events” in the second paragraph of the Abstract, the second paragraph of the Introduction section, the fourth and ninth paragraphs of the Results section, Table 3, and the first, third, and fourth paragraphs of the Discussion section as requested.


- Line 213 and 223 - “mainly” is inappropriate, an exact description of what was analysed should be reported.
Response: Thank you for your suggestion. We have deleted “mainly” from the sixth and seventh paragraphs of the Results section and the fifth paragraph of the Discussion section accordingly.

- Incidence of intubation needs to be reported even if not statistically significant.
Response: Thank you for your suggestion. We have presented the incidences of intubation in both arms in the eighth paragraph of the Results section.

DISCUSSION
- Line 259 – “lower” not “low” incidence.
Response: Thank you for your suggestion. We have revised this word accordingly.

- It is crucial that a definition of hypoxaemic event be defined. Without this, the discussion and indeed the study cannot provide a meaningful contribution.
Response: Thank you for your suggestion. We have defined “hypoxemic events” in the second paragraph of the Abstract, the second paragraph of the Introduction section, the fourth and ninth paragraphs of the Results section, Table 3, and the first, third, and fourth paragraphs of the Discussion section as requested.

- Line 267 – “respiratory deficits” is unusual wording, consider rephrasing and being more specific.
Response: Thank you for your suggestion. We have replaced the words in the second paragraph of the Discussion section to be more specific.

- Line 270 – what evidence is there that HFNC improves “bronchial hygiene” and what does this mean? Whole paragraph requires references for each physiological effect.
Response: Thank you for your suggestion. We have referred to PMID: 30725265, PMID: 25498581, PMID: 25742321, PMID: 27856714, and PMID: 26329355 and have revised the third paragraph of the Introduction as requested.

- Line 276 – “hazardous hypoxic events” – definition needed.
Response: Thank you for your suggestion. We have defined “hypoxemic events” in the second paragraph of the Abstract, the second paragraph of the Introduction section, the fourth and ninth paragraphs of the Results section, Table 3, and the first, third, and fourth paragraphs of the Discussion section as requested.

- Line 294 – this is not correct. Based on your analyses, it cannot be concluded that HFNC reduces CO2 clearance.
Response: Thank you for your suggestion. We have modified the fifth paragraph accordingly.

- Paragraph starting line 300 – additional limitation by population of lung transplant data which likely heavily influence results given baseline morbidity.
Response: Thank you for your suggestion. We have expanded the sixth paragraph of the Discussion section and have utilized sensitivity analysis to manage the resulting heterogeneity in the supplementary material (S3 Appendix).

- Limitations – elaboration needed on the small sample size and how this affects interpretation of results and clinical application. Define “acceptable oxygenation” and why this is an important point in the context of this review.
Response: Thank you for your suggestion. To make our manuscript more reasonable and coherent, we have elaborated on the influence of the small sample size using trial sequential analysis in the fourth paragraph of the Results section and have revised the seventh paragraph of the Discussion section according to the reviewer’s comment.

CONCLUSION
- This conclusion cannot be drawn based on the presented data.
Response: Thank you for your suggestion. We have revised our conclusion according to the reviewer’s suggestion.

- Needs to be much more comprehensive, cover the primary and secondary objectives, summarise limitations and provide suggestions to improve the quality of current evidence base.
Response: Thank you for your suggestion. We have revised the entire Discussion section and have provided references and suggestions in the fourth paragraph of the Discussion section according to the reviewer’s comment.

Acknowledgement – what editing was done?
Response: Thank you for your suggestion. We acknowledge Editage Academic Editing for editing this manuscript after the major revision.

Reviewer #4: Dear authors,
This is an interesting article and addresses an important issue (hypoxemia during bronchoscopy). I have following comments for the manuscript.
1. The statement “Clearly reported patient inclusion and exclusion criteria, medical treatment regimens, severity among the population, and the definition and evaluation of hypoxemic events during or after bronchoscopy were included in the analysis.” is not clear. Please rephrase it to clarify.
Response: Thank you for your suggestion. We have added information on the PICO selection to the first paragraph of the Methods section per your request.

2. In CONSORT diagram – Records screen 136 and irrelevant trial 105, the number should be 31, instead of 32.
Response: Thank you for your suggestion. We have updated our literature research published before August 2021, examined all numbers, and provided relevant information in the first paragraph of the Results section, Fig 1, and Supplementary material (S1) accordingly.

3. Please maintain uniformity in the figures – favour HFNC and favour COT should be consistently on same side. In figure 2 favour HNFC in right side whereas in figure 3 it is on other side.
Response: Thank you for your suggestion. We examined the direction of the forest plots carefully and confirmed that all directions were correct according to the Cochrane handbook (https://training.cochrane.org/handbook/current/chapter-10). We investigated the negative effect sizes in Fig 2 while we explored the positive effect sizes in Fig 3. Both figures show that HFNC was more effective in guaranteeing adequate oxygen than conventional oxygen therapy. Hence, Fig 2 should favor HFNC in the left side, and Fig 3 should favor HFNC in the right side.

4. English needs editing
Response: Thank you for your suggestion. We acknowledge Editage Academic Editing for editing this manuscript after the major revision.

5. Authors should also highlight that there is still debate over routine use of oxygen supplementation during bronchoscopy. Even at some centres, oxygen is only given to patients who have oxygen saturation of less than 96%.
Response: Thank you for your suggestion. We have added this limitation to the seventh paragraph of the Discussion and have provided the reference accordingly.

6. Authors, did not comment whether is any risk group which would get more benefits with HFNC.
Response: Thank you for your suggestion. We have elaborated on high pulmonary risk patients in the fourth paragraph of the Discussion section. Given that there are insufficient trials regarding HFNC in high pulmonary risk patients, we did not make conclusive results to make our manuscript tempered.

Reviewer #5: Comments to Author:
I think this study is highly novel regarding whether conventional oxygen therapy (COT) or high flow nasal canula (HFNC) is more effective in preventing hypoxemia for patients with during receiving bronchoscopy. However, there are several comments for this study. I am sorry, but I haven't read any further than the results, as I think there are serious problems with the methodology.
Major points
1. What is your research question? Is the presence or absence of sedation during bronchoscopy part of your question? I think the research question needs to be reconsidered because the presence of sedation is considered very important in the development of hypoxemia. In addition, the presence or absence of sedation can be a source of very large conceptual heterogeneity. Therefore, as I will point out below, I think it is highly likely that the search formula, inclusion and exclusion criteria will need to be reconsidered.
Response: Thank you for your suggestion. We have added information on sedation to the second paragraph of the Results section and to Table 1 accordingly.

2. Line 62-: How often is sedation used during bronchoscopy? If you want to discuss about hypoxemia during bronchoscopy associated with sedation, I think the frequency of sedation needs to be described.
Response: Thank you for your suggestion. We have provided the duration and methods of sedation in Table 1.

3. Line 67-: What exactly does “debilitating” mean? Have adverse events associated with hypoxemia been discussed in previous studies? If you want to express the need for oxygen administration, I think it would be more persuasive if you describe a specific description of the serious adverse events associated with hypoxemia.
Response: Thank you for your suggestion. We have cited the following studies (PMID: 30725265, PMID: 25498581, PMID: 25742321, PMID: 27856714, and PMID: 26329355) and have revised the first paragraph of Introduction section accordingly to avoid ambiguity.

4. Line 77-: In this paragraph, I think it is necessary to subscribe, for example, that recommendations may change because multiple randomized controlled trials (RCTs) have been reported but no systematic review (SR) or meta-analysis (MA) have not been integrated done. It seems to me that you are now describing why we must do RCTs.
Response: Thank you for your suggestion. We have revised the third paragraph of the Introduction section accordingly.

5. Line 95-: If your research question includes the presence or absence of sedation during bronchoscopy, then I think you need to add the presence or absence of sedation to your inclusion or exclusion criteria.
Response: Thank you for your suggestion. First, large irrelevant trials regarding surgery and anesthesia may be included if we involve sedation. Second, eligible trials must include the term “bronchoscopy.” Therefore, we did not change our search strategy to ensure that our PICO search remains precise.

6. Line 101-: Have you done any searches about ongoing studies? In the methods section, the authors stated that ClinicalTrials.gov registry was searched for ongoing trials. However, there was no results of the search in result section. Please explain this point.
Response: Thank you for your suggestion. We have updated with relevant trials published before August 2021 and have provided the search strategy in the first paragraph of the Results section, Fig 1, and supplementary material (S1) accordingly.

7. Line 103-: Is it correct to assume that the following search formula was used: “HFNC” OR “high flow nasal cannula” OR “high flow nasal CPAP” OR “high flow nasal oxygen” AND “bronchoscopy” OR “bronchoscope”. Is this search term sufficient for the search formula? First, it is true that there is no MESH term of HFNC, but bronchoscopy of MESH term. When you search with your search formula, you cannot find all the synonyms, plurals, etc. contained in the MESH term. Second, If the search is to be done as you intend, then I think the following use of () is correct: (“HFNC” OR “high flow nasal cannula” OR “high flow nasal CPAP” OR “high flow nasal oxygen”) AND (“bronchoscopy” OR “bronchoscope”). Third, if you are targeting an RCT, you should use the study design filters which Cochrane recommended. I think that your current search formula is inadequate and does not allow you to perform the correct search. If you can't do the correct search, SA and MA will be completely useless.
Response: Thank you for your suggestion. Although there were no MeSH terms regarding high flow nasal cannula, we indeed reviewed other meta-analyses and examined the Emtree carefully. The words of high flow system must include “HFNC” OR “high flow nasal cannula” OR “high flow oxygen” OR “high flow nasal oxygen” unless they did not perform the study regarding HFNC. Besides, we also searched the bibliographic databases for a thorough, objective, and reproducible search strategy. First, we checked the keywords and MeSH terms on PubMed and Embase (Emtree) to ensure that no relevant trials were missed. Second, we merged the keywords and MeSH terms manually in the Cochrane library because this database does not provide a link between keywords and MeSH terms. Third, we examined the titles and abstracts and retrieved the full text of the eligible trials according to the inclusion and exclusion criteria. Fourth, we assured that the relevant trials were in compliance with the PICO elements and made the final decisions on inclusion. Fifth, the terminology of HFNC was usually presented as “high flow nasal cannula” and “high flow nasal oxygen.” Fifth, we did not restrict the search based on language and publication years, add any quotes and truncation and restrict the PICO elements to the title and abstract. Therefore, using this strategy, we could theoretically locate all relevant articles, unless the trials were not concerned with HFNC in patients undergoing bronchoscopy. Because we did not set any restrictions, we increased the power of the results and the generalizability of the findings. Eventually we enrolled five RCTs in our meta-analysis. To clarify these issues, we have provided a description of our search strategy in the first paragraph of the Results section, Fig 1, and supplementary material (S1) accordingly.

8. Line 111-: I think it is necessary to extract and consider the procedure time for bronchoscopy. There is a significant correlation between the procedure time for bronchoscopy and the presence of hypoxemia. If it is not mentioned in each study, then I think it should be mentioned as such in your study.
Response: Thank you for your suggestion. We have provided relevant information in the second paragraph of the Introduction and in Table 1 accordingly.

9. Line 125-: What exactly is the definition of “the incidence of hypoxic events during bronchoscopy” in your study? Is it correct that you consider the outcome of each of the RCTs included in this MA as "the incidence of hypoxic events during bronchoscopy"? I think PaCO2 at the
end of bronchoscopy and EtCO2 at the end of bronchoscopy are different from “the incidence of hypoxic events during bronchoscopy”. After establishing specific clinically meaningful outcomes for hypoxemia, you should decide whether you can integrate the results of each study.
Response: Thank you for your suggestion. First, we have defined “hypoxemic events” in the second paragraph of the Abstract, the second paragraph of the Introduction section, the fourth and ninth paragraphs of the Results section, Table 3, and the first, third, and fourth paragraphs of the Discussion section. Second, we rechecked the Methods section, Results section, and tables to verify the timing of hypoxemic events (SpO_2_ < 90%). Third, we considered that PaCO_2_ and EtCO_2_ were important for respiratory system assessment. It was ideal for us to present the date during the bronchoscopy, but Longhini and Lucangelo et al. only provided the values at the beginning, at the end of bronchoscopy, and after the procedure. Hence, it was a limitation that precluded a more precise analysis. We have expanded the seventh paragraph of the Discussion section to clarify this issue.
